# Point-of-care C reactive protein for the diagnosis of lower respiratory tract infection in NHS primary care: a qualitative study of barriers and facilitators to adoption

**DOI:** 10.1136/bmjopen-2015-009959

**Published:** 2016-03-03

**Authors:** Jeremy R Huddy, Melody Z Ni, James Barlow, Azeem Majeed, George B Hanna

**Affiliations:** 1 Department of Surgery and Cancer, Imperial College, London, UK; 2 Imperial College Business School, South Kensington Campus, London, UK; 3 Department of Primary Care and Public Health, Imperial College London, London, UK

**Keywords:** QUALITATIVE RESEARCH, PRIMARY CARE

## Abstract

**Objectives:**

Point-of-care (POC) C reactive protein (CRP) is incorporated in National Institute of Health and Care Excellence (NICE) guidelines for the diagnosis of pneumonia, reduces antibiotic prescribing and is cost effective.

**Aim:**

To determine the barriers and facilitators to adoption of POC CRP testing in National Health Service (NHS) primary care for the diagnosis of lower respiratory tract infection.

**Design:**

The study followed a qualitative methodology based on grounded theory. The study was undertaken in 2 stages. Stage 1 consisted of semistructured interviews with 8 clinicians from Europe and the UK who use the test in routine practice, and focused on their subjective experience in the challenges of implementing POC CRP testing. Stage 2 was a multidisciplinary-facilitated workshop with NHS stakeholders to discuss barriers to adoption, impact of adoption and potential adoption scenarios. Emergent theme analysis was undertaken.

**Participants:**

Participants included general practitioners (including those with commissioning experience), biochemists, pharmacists, clinical laboratory scientists and industry representatives from the UK and abroad.

**Results:**

Barriers to the implementation of POC CRP exist, but successful adoption has been demonstrated abroad. Analysis highlighted 7 themes: reimbursement and incentivisation, quality control and training, laboratory services, practitioner attitudes and experiences, effects on clinic flow and workload, use in pharmacy and gaps in evidence.

**Conclusions:**

Successful adoption models from the UK and abroad demonstrate a distinctive pattern and involve collaboration with central laboratory services. Incorporating antimicrobial stewardship into quality improvement frameworks may incentivise adoption. Further research is needed to develop scaling-up strategies to address the resourcing, clinical governance and economic impact of widespread NHS implementation.

**Strengths and limitations of this study**

- Increases the understanding of the barriers to widespread adoption of point-of-care C reactive protein in National Health Service primary care to guide the prescription of antibiotics for lower respiratory tract infection.
- Informs the development of mitigating strategies to overcome these barriers, including examples of where this has been achieved in Europe.
- Contributes to the growing body of evidence to support the adoption of the testing strategies particularly given the widespread recognition of the importance of tackling antimicrobial resistance and reducing inappropriate prescribing of antibiotics.
- The study included a variety of stakeholders, allowing for an overall prospective of potential barriers but at the expense of the sample size in each role.
- The nature of the research may introduce recruitment bias, as stakeholders with favourable attitudes towards the test may be more likely to accept invitations to participate.

## Introduction

The majority of patients presenting to primary care with a suspected lower respiratory tract infection (LRTI) are prescribed antibiotics.^1^ However, most respiratory tract infections are viral and only marginal benefit is achieved from the prescription of antibiotics that in some cases does not outweigh the risk of harm.^2–4^ One of the key aims of the UK 5-year Antimicrobial Resistance Strategy is to conserve and steward the effectiveness of existing treatments. Point-of-care (POC) C reactive protein (CRP) testing for patients with suspected LRTI has been included in guidelines in Norway, Sweden, the Netherlands, Germany, Switzerland, Czech Republic and Estonia to determine severity of infection and extent of inflammation. In the Netherlands, specific algorithms have been developed to guide antibiotic prescribing using CRP thresholds.^5^ Current devices allow CRP testing to be performed from a finger-prick sample and analysed in approximately 4 min. This strategy is recommended by European Respiratory Guidelines.^6^ Several studies^7–9^ and a Cochrane Review^4^ have demonstrated reduced antibiotic prescribing as a result.

In 2014, CRP testing was incorporated in the National Institute of Health and Care Excellence (NICE) guidelines for the diagnosis of pneumonia.^10^ The guidelines recommend not routinely offering antibiotic therapy if the CRP is <20 mg/L, considering a delayed antibiotic prescription if CRP is in the 20–100 mg/L range, and offering antibiotic therapy if CRP concentration is >100 mg/L. There is widespread recognition of the importance of tackling antimicrobial resistance, and reducing antibiotic prescribing will not only improve antibiotic stewardship but also offer a significant financial saving for the National Health Service (NHS); two recent cost-effectiveness studies have demonstrated the potential cost savings of POC CRP in the management of LRTI.^11^ ^12^

Previous qualitative studies have suggested that providing a support tool such as POC CRP to general practitioners (GPs) would be received positively, and has the ability to improve the consultation by highlighting disease severity, manage patient's expectations, and increase confidence in antibiotic prescribing.^13–15^ However, despite POC CRP being routinely used in many European countries, the test is yet to receive mainstream adoption in NHS practice. This study aimed to identify the barriers to adoption of POC CRP testing in NHS primary care, and how similar challenges were overcome in European countries where POC CRP use is now widespread.

## Methods

A qualitative study was undertaken in two phases based on grounded theory. The study description and results are summarised in accordance with the Consolidated Criteria for Reporting Qualitative Research (COREQ) checklist.^16^ Stage 1 was a semistructured interview study that took place in February and March 2015. Early adopters in the use of POC CRP were recruited based on their involvement in clinical trials relating to POC CRP and identified either from our literature search or following a recommendation by Alere International who funded the study. Participants were recruited from both European countries where POC CRP has already been widely adopted, and from UK NHS practice. Convenience sampling was undertaken, and potential participants were included based on their experience in integrating POC CRP into clinical practice. Invitations to participate in the study were distributed by email.

The interviews were undertaken by one author (JRH) who is a male clinical research fellow at Imperial College with experience in qualitative research gained through the ongoing development of the Point-Of-Care Key Evidence Tool (POCKET);^17^ there were no non-participants present during the interviews. The interviewer did not know any of the interview participants before the investigation, and the participants received information on the interviewer, background and aims of the research project before the interview. The interviews were semistructured, and a list of prepared topics and questions were prepared by two authors (JRH and JB), piloted, and used as a prompt (see online supplementary appendix 1). Interviews were undertaken by telephone or Skype and recorded with no field notes made. Following verbatim transcription, transcripts were not returned to participants unless clarification was required and no repeat interviews were undertaken. Interviews were analysed by two researchers (JRH and MZN, a female Senior Research Fellow at Imperial College with a background in Decision Sciences and the application of Decision Analysis to medicine) who independently reviewed the transcripts using constant comparative techniques before meeting to compare emergent themes. Interviews were undertaken until saturation had been reached, as demonstrated by the absence of new themes emerging from analysis.

10.1136/bmjopen-2015-009959.supp1 Supplementary appendix

Stage 2 was a 2 h facilitated group workshop on 30 March 2015, and focused solely on the barriers to NHS adoption. An invited multidisciplinary group of stakeholders in the adoption of POC-in-vitro diagnostic (IVD) were invited. Relevant stakeholder groups were identified from the interview study and included GPs (including those with commissioning experience), biochemists, pharmacists, clinical laboratory scientists and industry representatives. Convenience sampling was undertaken. GPs were recruited through the Department of Primary Care and Public Health at Imperial College London, and other stakeholders directly recruited by the NIHR Diagnostic Evidence Co-operative. All invitations were sent by email. A maximum group size of 16 was selected to allow for a round table discussion format. A fee was paid to the facilitator, and an honorarium was made to the practices of primary care participants to compensate for their clinical time.

Two presentations preceded the workshop; the first outlined current evidence for POC CRP including economic analyses, and the second presented the emergent theme analysis from the interview study. Facilitated discussion then covered barriers to adoption, impact of adoption and adoption scenarios. The workshop facilitator was external to the research group to minimise bias but did have expertise in the use of POC CRP. Three members of the research group attended the workshop including a clinician, decision analyst and member of faculty from the Imperial College Business School who independently reported on emergent themes from the workshop. The workshop was also recorded and transcribed for analysis in the same way as the interviews and participants were not asked to provide further feedback on findings.

All interview and workshop participants had provided informed consent to participate in the study. Qualitative data was analysed with NVivo V.10.1.1 software (QSR International, Melbourne, Australia).

## Results

Eleven experts in POC CRP were invited to participate in the stage 1 interview study, of whom eight agreed. Two participants declined due to lack of availability during the study period and one participant did not respond to the invitation. Interviews had a mean length of 27 min (range 20–38). Four interviews were carried out with practicing UK GPs, two of whom had an academic background. The other four interviews were carried out with practitioners from European countries where POC CRP is widely used, with one each from Denmark, Norway, the Netherlands and Sweden, all of whom were active in research.

In stage 2, 24 participants were invited to attend the workshop of whom 10 attended; reasons for non-attendance at the workshop were lack of availability (10), no response from invitation (2) and acceptance of invitation but subsequently not attending (2). The stakeholder breakdown of workshop attendees was industry representatives (3), GPs (2) including one with experience as a clinical commissioning group board member, biochemist (1), clinical laboratory scientist (1), pharmacist (2) and primary care research manager (1). Six of the workshop participants were men. Seven themes were derived from the interviews and workshop transcripts regarding the barriers to adoption of POC CRP in the NHS: reimbursement and incentivisation, quality control and training, laboratory services, practitioner attitudes and experiences, effects on clinic flow and workload, use in pharmacy and gaps in evidence. The positive and negative incentives for investing in the adoption of POC CRP vary between stakeholder groups and mitigating strategies are summarised in table 1.

**Table 1:** Summary of incentives and disincentives for POC CRP adoption by stakeholder group

Stakeholders	Reasons FOR adoption	Reasons AGAINST adoption	Recommendations/next steps
NHS	Early intervention by GP; less hospital referrals; NICE pneumonia guidelines recommendations; evidence of reduction in unnecessary antibiotics prescription when CRP test results are used	Funding mechanism needs to balance encouraging adoption of POC CRP and overuse	Clear POC CRP user guideline necessary
General Practitioners	Increased diagnostic confidence; increased decision-making support especially when in doubt; improved communication with patients; improved access to test that overcomes geographical distance; early adopters/opinion leaders continue to publish evidence that favours wider-scale adoption	Behaviour inertia and risk aversions; perception of the test taking up too much time; POC CRP expensive to take up	Perception of time can be corrected—successful adoption model exists; further evidence required on clinical utility at individual GP practice
Laboratories	Active role in quality control, training, maintenance of POC devices that are consistent with the future of laboratory services	Income loss due to not performing CRP tests, but the impact presumably small	Funding route carefully managed to encourage and ensure quality maintenance role by clinical laboratories

CRP, C reactive protein; GP, general practitioner; NHS, National Health Service; NICE, National Institute of Health and Care Excellence; POC, point-of-care.

In what follows, we present issues identified from the interviews and workshop that would facilitate or impede the adoption of POC CRP testing into NHS General Practice in respect to the management of LRTI. A summary of adoption models from the European countries where use is now mainstream is also included.

### Reimbursement and incentivisation

Primary care practices cannot use the test unless it is properly resourced. Across Europe, different reimbursement structures exist with associated benefits and risks. Models from Europe include direct cost reimbursement through insurance companies or government, government payments for performing the test above direct test cost, device provision by commercial laboratory services as part of block contracts.

However, it was highlighted that some reimbursement systems may encourage inappropriate use or overuse. Linking reimbursement programmes to clinical guidelines may mitigate this if payment is only made when the test is performed according to guidelines. Most participants see POC CRP as a cost-effective intervention but concerns were highlighted that inappropriate use may counteract any financial savings.

Several procurement options were proposed throughout the study for the NHS. These included:

- Direct purchase by the primary care practice;
- Purchase by a clinical commissioning group to cover practices in their region;
- Block purchase by central government bodies (eg, NHS England, or Public Health England) on a national level;
- Purchase and ownership of devices by central laboratory services and loaned/leased to primary care practices (with the option of including service contracts to cover quality assurance, maintenance and training);
- Loan/lease agreements from industry (with the option of including service contracts to cover quality assurance, maintenance and training).

Overall, to facilitate NHS adoption, GPs will need to be incentivised to adopt the technology. The recommendation of POC CRP use in recent NICE guidelines should be an initial facilitator for change, but the support of other organisations is required to drive this. In Europe, general practice colleges have led this through the development of guidelines and algorithms for recommended use. This may be a viable option in the UK but it was felt that a stronger drive would be POC CRP testing, and antimicrobial stewardship being built into primary care quality improvement programmes and the Care Quality Commission setting out its use as an expectation. Public health policy was previously under the remit of the primary care trusts which were answerable to strategic health authorities. This responsibility now rests with local authorities with indices and metrics less aligned to clinical practice, and therefore, a public health policy approach may require a national intervention. The availability of test device platforms that can perform other tests (eg, glycated haemoglobin (HbA1c) or lipids) was seen as an additional incentive (box 1).

Box 1 **Direct quotations from participants relating to reimbursement and incentivisation**

“Getting the reimbursement formula right is important…it shouldn't just be, I think, use it every time you like and you'll get paid for it…but primary care can't do it unless it's properly resourced.” (Interview participant 6)

“The costs would need to be reimbursed in some format, and it would need to be part of a quality improvement programme for general practice.” (Interview participant 5)

“It's widely used in Scandinavian countries because the clinicians get basically a payment each time they use the test.” (Interview participant 6)

“The problem is, if you paid them [GPs] every time they use it, then they're going to use it when they don't really need to be using it.” (Interview participant 6)

“Some sort of quality standard that includes CRP testing within a fairly rigid protocol, which is able to be audited and reported might be a beneficial process, although it would need to be not open to abuse.” (Interview participant 5)

“The CCG would need to make a decision…we're going to put these machines in every practise and pay for them…I'm quite certain that GPs wouldn't just do this out of their own pockets because that's just not the way it works.” (Interview participant 4)

“The engagement needs organisations, such as NHS England and CQC…making directives that practices have got to have this equipment by such and such a date, CQC, in their regulatory role, making sure that things are put in place, and making it known in the annual cycles of change, that this is the sort of thing they're looking for, so that come this time next year, practices have got this in place.” (Workshop participant)

“To get people, particularly in terms of path finding, or innovating, or piloting different areas, you tend to have to incentivise them.” (Workshop participant)

“Maybe general practitioners will be told they have to finance it themselves from their income, but again, that would need CQC to have the support of NICE, to say this is an essential expectation of all general practise and all primary care.” (Workshop participant)

### Quality control and training

The responsibility for quality control will either belong to primary care practitioners themselves or be outsourced to external bodies. European examples exist of both local and national external quality control organisations. Maintenance of devices, quality control, and an awareness of the shelf life of test cartridges will be essential components of such a system. Devices should also offer connectivity so that results can be uploaded to local patient information systems.

Participants were aware of the responsibilities laid down by the Medicines and Healthcare products Regulatory Agency^18^ when adopting POC technologies and the associated cost of quality assurance raises concerns. Potential mitigating strategies included service contracts provided by industry to undertake these responsibilities, as seen in some examples of POC being used for health check programmes, or ownership of quality control responsibilities being allocated to central laboratory services that already have processes in place to validate technologies, keep asset registers and adhere to appropriate governance requirements.

One particular governance issue that was repeatedly discussed was training both in performing the test and the clinical application of results for practitioners. Training programmes can be delivered by industry, central laboratory services, in-house or by online video training. Some European reimbursement schemes incorporate evidence of training as a pre-requisite for reimbursement. Training requirements will need to be adapted to different GP practices based on the size of the practice and who will be performing the test. It was felt that short refresher training would also be required, given the possible gaps in use due to the seasonal nature of the test (box 2).

Box 2 **Direct quotations from participants relating to Quality Control and Training**

“I want to make a strong plea for quality assurance, and that GPs should prove that they attach to such a programme.” (Interview participant 2)

“If this could be devolved with the CCG, whereby the CCG can have an asset list, it removes some of the red tape, from a GP perspective.” (Workshop participant)

“The lab [central laboratory services] would be able to help with that…we already have existing systems in place to meet the MHRA standard, the CQC standard for point of care…we already have an asset register…so all those governance issues, you won't have any problem with that.”

“I think this is going to be part and parcel of the whole process of accreditation, quality assurance, incentivisation, training, audits, it's all part of quality control.” (Interview participant 5)

“Respiratory tract infections being seasonal, one could imagine a long period of time when the equipment is not used…what are the risks of inaccuracy with equipment when one is not familiar with using it.” (Workshop participant)

“Any training packages have got to have different modalities between face-to-face group training, videos, refresher videos, video manuals, or things which can be quite short as refreshers, maybe just a minute or two for the slightly overworked and oppressed clinician trying to make a decision on a patient late at night, as opposed to the novice using it for the first time.” (Workshop participant)

### Laboratory services

All POC CRP users from Europe and UK have good working relationships with proactive central laboratory services. Some participants speculated about the potential loss of income to central laboratory services, and how this might represent a barrier to adoption of POC, but there were no cited examples of this. Competition with central laboratory services was therefore not thought to be an issue. This is partly because users of POC CRP rarely duplicated CRP testing by sending samples to central laboratories in addition to POC results. In addition, when POC testing is not available, it is unlikely that a sample would be taken for central laboratory analysis, as the result would not alter decision-making during the consultation. Where POC CRP testing is undertaken, it was always in partnership with proactive central laboratory services or community POC committees assisting with quality assurance, stocktaking, and the monitoring of training. This collaboration also permits the linking of results to patient electronic results systems and to electronic patient records. There were several examples of laboratories providing POC CRP devices to GP practices with service contracts as part of block service contracts. A concern with this model is that laboratories may prefer to install POC devices in busy inner-city practices, where they will undertake more tests and see a faster return on their investment, disadvantaging rural practices (box 3). Easy access and quick results turnover availed by the POC device is particularly beneficial when a laboratory is geographically distant or when it is out of service (eg, out of working hours).

Box 3 **Direct quotations from participants relating to Laboratory Services**

“What helps the implementation is the fact that we've got a dedicated point-of-care team central laboratory and the chemistry laboratory who will come out, plug in and teach the standard operating procedures.” (Interview participant 8)

“Perhaps that is where the pathology department can help support the GP. You say you can't do technical evaluation in the patient group that you will actually will get…we do that routinely within the hospital.” (Workshop participant)

### Practitioner attitudes and experiences

Feedback from POC CRP users was positive, with practitioners reporting increased reassurance with their management plans, increased job satisfaction, and an improvement in the ‘diagnostic pleasure’ derived from incorporating the test result into decision-making. However, the participants described the perception of a ‘point-of-care block’ existing in primary care. Anecdotal examples of resistance to POC included GP colleagues believing that extra tests, such as CRP, were not necessary to make a diagnosis; that undertaking the test leads to an unacceptable increase in duration of patient consultation; and concerns about the sensitivity of the test and the appropriate way to manage an unexpected or misleading rise in CRP when the clinical findings do not correlate.

Many GPs regard the test as an adjunct to their communication when educating patients in antimicrobial stewardship. However, one GP raised concerns that increased reliance on diagnostic tests may restrict the holistic approach that is fundamental to primary care patient interactions, and psychosocial aspects of a patient's presentation may be missed. The participants who had experience in POC CRP use all highlighted positive reactions from patients in relation to the test. One GP simply answered ‘They love it’. No cultural barriers were identified in the study, although there were concerns that the popularity of the test may predispose to overuse both arranging GP consultations with minor symptoms to benefit from the ‘reassurance’ of a normal CRP, or sequential testing following a normal or borderline result if symptoms were not resolving (box 4).

Box 4 **Direct quotations from participants relating to Practitioner attitudes and experiences**

“Changing clinician behaviour is a slow process and there's an organic and a cultural process.” (Workshop participant)

“Instinctively, GPs feel that they don't need a lot of tests to make a diagnosis.” (Interview participant 4)

“It's almost like…they [GPs] feel that the machine will make the decision on behalf of them…it deprives them of the decision making capability, the learning opportunity.” (Workshop participant)

“Anything that prolongs the consultation will be seen as a problem.” (Interview participant 4)

“GPs are very satisfied by having an instrument in the consultations that can help them to make the right decision.” (Interview participant 3)

“If we set up a facility, which offers near patient testing, numericising an illness, or a future of an illness, there's a risk we become very much transactional doctors…and we've always prided ourselves in primary care that we maybe discriminate some of the psychosocial symptoms and de-medicate our patients better that our secondary care colleagues. I'm not saying that that outweighs the benefits at all, but I think it needs to be taken into account.” (Workshop participant)

“It's quite useful when they say they might feel the person doesn't need antibiotics but the patients are keen for the antibiotics. Then it's an adjunct to the communication skills.” (Interview participant 6)

“May be that people want to come back because they love the test so much.” (Interview participant 4)

“They've got CRP of about 20, 18 or something like that. But two days later, they're not very well, so they'll want another CRP…this is opening a potential can of worms.” (Workshop participant)

“Reassurance can be a very powerful tool.” (Interview participant 4)

“I think it increases the trust in the GP.” (Interview participant 1)

“Nowadays patients, if they have a sore throat or they have catarrh they may think I have to go to the doctor not to get antibiotics but to have the test.” (Interview participant 3)

### Effects on clinic flow and workload

**Figure 1:**
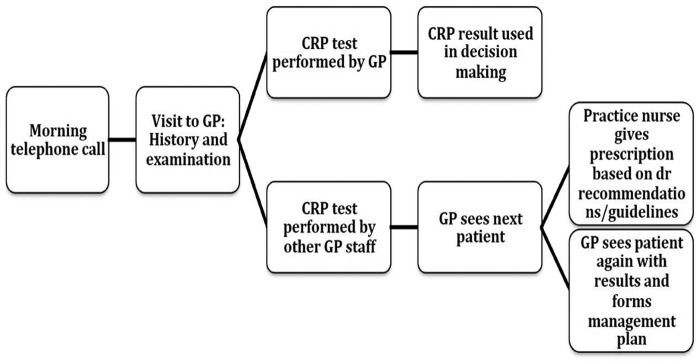
Flow diagram of typical patient pathway incorporating point-of-care CRP testing. CRP, C reactive protein; GP, general practitioner.

Three participants discussed their impression of the impact of POC CRP testing on clinic flow. One described no effect, one described a change in flow with no overall consequence, and the final one observed that there is an effect but a robust process for testing can mitigate this. GP workload is currently at capacity,^19^ and any perceived effects on clinic flow, additional workload, or increasing patient consultations may inhibit adoption of the test. However, the impact on workload will depend on the approaches to testing adopted by specific GP practices. Some examples were given of the test being undertaken by the GP within the consultation, others included the patient leaving the consultation to have the test being undertaken by a practice nurse, healthcare assistant, or inhouse primary care laboratory technician, while the practitioner sees the next patient; decision-making would then follow based on written instructions from the practitioner, or a second consultation being undertaken. These options are summarised in figure 1 (box 5).

Box 5 **Direct quotations from participants relating to Effects on clinic flow and workload**

“It will have an effect, but if you have the healthcare assistant do the test, while you see another patient, it shouldn't make too much difference.” (Interview participant 6)

“I think most doctors will see another patient in between and return to the first patient afterwards.” (Interview participant 2)

“I think we've got barriers of the workload, and the workload in clinical practise is very heavy at the moment. It's, sort of, recognised on a national level. And capacity comes indifferent areas, not just the capacity of workload, it's the capacity and space.” (Workshop participant)

“Once patients say ‘that was a really good test doctor…I want the test’…they are entitled to the service. Now, they don't want the antibiotics, they want the test. It's about managing demand. Unfortunately, even with infinite capacity, I promise you, where cost is free, you'd have infinite demands as well.” (Workshop participant)

“If it's an infinite resource, you could argue that you could do this point of care testing on everyone who presents with a cold. Where do you draw the line?” (Workshop participant)

“There are clear-cut cases you think we should prescribe [antibiotics]. Maybe it's the middle area that you find it hard to convey to patients…and you have that additional thing to help to convince, actually, that it's a good idea not to give antibiotics.” (Workshop participant)

### Use in pharmacy

Many patients with symptoms of LRTI do not present to their GP. Community pharmacies were highlighted as a potential alternative setting for POC CRP. As with primary care surgeries, this would need appropriate incentivisation, particularly as pharmacists may lose income if antibiotic prescribing is reduced. NHS initiatives are encouraging pharmacist independent prescribers in certain areas such as hypertension and respiratory illness,^20^ ^21^ and it was suggested that antibiotic prescribing for LRTI, guided by a POC CRP-based algorithm, could be evaluated for inclusion in future policies. Some concern was raised that this may duplicate work, as patients may arrange a GP visit in addition to pharmacist consultation, and a significant culture change may be required to negate this (box 6).

Box 6 **Direct quotations from participants relating to where further evidence is required**

“We need the evidence, we need the evidence across all areas, not just antibiotic resistance, but…in terms of clinical outcomes, that this is an effective tool in delivering improved healthcare.” (Workshop participant)

“I think that's the kind of space for implementation study that might still be needed.” (Interview participant 6)

### Where further evidence is required

The evidence for POC CRP is summarised in the introduction. However, gaps raised during the interview study included the need for an implementation study to investigate overall patient and societal outcomes and alternative indications for POC CRP in primary care. Workshop participants also sought further evidence on the risks of not prescribing based on POC CRP and guidance in the application of test results to different populations, including children and patients with comorbidity (such as rheumatoid arthritis and chronic obstructive pulmonary disease).

### Adoption models in Europe

Successful adoption models in the European countries where we interviewed experts showed a distinct pattern. That is, a slow and long early adoption phase followed by policy changes that then triggered large-scale adoption. In countries where the device is widely used, a few opinion leaders became early adopters, recognising the importance of using the device to support their decision-making in antibiotics prescriptions. Through clinical practice and research, these early adopters continued to generate and publish evidence. Eventually, the national professional bodies became advocates of the new technology. The accumulation of evidence and support from the practitioners, especially when embedded in a policy environment that encouraged the use of POC technology and a government drive to reduce over-prescription of antibiotics, laid the foundation for a resource allocation model that supported large-scale adoption. However, the process was regarded as slow, ranging from 2 to 7 years between initial adoption by opinion leaders and the eventual large-scale implementation.

## Discussion

### Summary of main findings

This qualitative study found a number of barriers that stand in the way to the mainstream adoption of POC CRP testing into NHS primary care for the management of LRTI. These challenges have been mitigated in other European counties where the test is more widely used, and lessons can be learnt from their practice. Many strategies could be directly transferred to UK practice and scaled-up to promote NHS implementation. The primary barrier at present is the absence of a UK funding and reimbursement model for the test. Several funding models were proposed during the study, each with their own incentives for reimbursement, but there is a risk that inappropriate schemes may lead to overuse or underuse of the test potentially leading to an overall reduction in cost-effectiveness.

There were concerns raised by practitioners regarding the burdens associated with maintenance, stocking and quality assurance of the test device and cartridges. It may be that as seen in Europe, laboratories can play a role in the process with resource allocation models to support laboratories to actively take part in primary care POC testing with service agreements to act as test administrators, quality controllers and trainers.

Implementation of POC CRP will require changes to working practices and clinic flow. The time and human resources required to undertake testing will need to be absorbed into already stretched clinics. Examples of how this has been achieved were included, but individual practices and practitioners will need to consider their own staffing, infrastructure and culture when establishing a testing service. While the study investigated the use in primary care, alternative models were proposed including use of the test in pharmacy. The increasing use of pharmacist prescribers will make this feasible, and many pharmacists are keen to offer such enhanced services.

### Strengths and limitations

This study does have several limitations. From the sample size we have, we may not have presented a comprehensive overview of POC CRP use and adoption across all of Europe. Instead, we aimed to select participants with academic backgrounds who were involved in the early stages of adoption in their respective countries to learn from their success. Second, as with all qualitative research, study participation involves a time burden to undergo interviews and attend workshops. Therefore, the study is prone to recruitment bias, as those with positive attitudes towards the test may have been more likely to attend, and there were many invited participants who choose not to participate, although data collected and presented includes positive and negative beliefs regarding the test. Every attempt was made to recruit a representative sample of stakeholders, but convenience sampling was unavoidable. We believe we have included a wide range of stakeholders with differing interests in the test use, and that the highlighted themes are of importance and relevance within the NHS as a whole. However, a tradeoff for including this variety of stakeholders was that the sample size in each role was reduced. In particular, only two GPs attended the workshop, and although previous research has been undertaken to investigate the attitudes of this group a larger sample of GPs would have benefitted this study. As included stakeholders were experts in the use of POC CRP, patients were not included in this study. Therefore, the reporting of patient attitudes was anecdotal and needs validation.

We used a workshop facilitator who was independent to the research group and a series of open-ended questions to minimise any bias resulting from the prior beliefs of the group. The researchers undertaking the coding were not from a primary care background but did have a clear understanding of the clinical pathway and included expertise in medical decision-making. By not sharing the professional background of the participants, there may be restrictions in the depth of exploration undertaken, but it was felt that the coders background meant they were less likely to have their own beliefs influence the study results, and coding was undertaken independently without a requirement for consensus. Finally, peer debriefing was not undertaken, although there was scope within the methodology to clarify any aspect of the collected data if it were to have been required.

### Comparison with existing literature

A previous systematic review of primary care practitioner attitudes to the use of POC tests highlighted concerns about accuracy, misleading results, over-reliance and the cost of equipment and maintenance and tests not being helpful in altering consultations.^22^ While many of the same apprehensions were discussed in the current study, most were not seen as barriers. Accuracy was discussed, but this was in respect to distinct patient groups, such as children or patients with comorbidities, where further evidence is required, and current POC CRP devices have been shown to perform with similar accuracy to laboratory analysers.^23^ The cost of the test was highlighted in respect to the need for a suitable reimbursement model, but the overall cost-effectiveness of test adoption was felt to be a facilitator. One focus group participant did raise the subject of over-reliance, and the risk of transforming the consultation into a paper exercise, potentially detracting from the holistic approach to what is often complex multifactorial patient care. However, a caveat was provided that the benefits of reducing antibiotic prescribing may outweigh these concerns, and when the test is used appropriately, the extra information provided should improve the quality of consultations both in respect to the doctor–patient relationship and the end management.

Overall practitioner attitudes towards the adoption of POC CRP were encouraging. The perceived benefits in antibiotic prescribing and communication were forthcoming, and of those stakeholders already using the test in clinical practice, patients’ responses were perceived to be positive. This coincides with a recent survey study that demonstrated that 61% of UK GPs would welcome access to POC CRP in their clinic,^24^ and studies have repeatedly demonstrated a high rate of patient acceptability towards POC testing strategies.^6^ ^14^ ^25^

### Implications for practice

Many barriers discussed in this paper are perceived and can be overcome with relatively minor changes to work patterns, culture and education. However, structural barriers including the lack of funding model and reimbursement strategy will require policy change if widespread NHS adoption is to be achieved. Within the UK, POC CRP has been recommended in the NICE pneumonia guidelines. Using CRP testing is also consistent with the government drive to reduce antimicrobial resistance. Given that GP practices may be initially unwilling to fund the device themselves, a successful funding mechanism that would lead to large-scale adoption would (1) facilitate take-up and implementation into mainstream practice, (2) discourage device misuse and (3) encourage active role transition played by the clinical laboratory. It is essential that clear guidelines exist to govern how and when POC CRP testing should be used, and establishing antimicrobial stewardship into quality improvement frameworks may achieve this.

In conclusion, many GPs would welcome POC CRP as an additional tool for their diagnostic armoury. Disincentives and incentives exist for all stakeholders, but mitigating strategies exist. Further research should be aimed at investigating the impact of scaling-up NHS implementation at a patient practice and wider health system level. Use of the test in children and those with comorbidities should also be investigated. Particular focus needs to assess the economic impact of test adoption in primary care to ensure the cost-effectiveness described in previous reports^7^ ^8^ holds true in real-world adoption.
