# Supplementary material for: Point-of-care C reactive protein for the diagnosis of lower respiratory tract infection in NHS primary care: a qualitative study of barriers and facilitators to adoption
Source: BMJ Open. 2016 Mar 3;6(3):e009959. doi: 10.1136/bmjopen-2015-009959 (PMC4785316; doi:10.1136/bmjopen-2015-009959)
Supplement: Supplementary appendix [file bmjopen-2015-009959supp.pdf]

Appendix 1.

<u>Topic prompt for stage 1 interviews.</u>

Overview of local healthcare system (for international interviews)

- Please may you describe your role in your healthcare system?
- How does primary care interact with secondary care?
- Is point-of-care testing in primary care part of routine practice?
- Do patients expect POC tests when visiting their GP? Are POC test routinely performed in primary care for other diseases? What diseases?
- Are POC tests regulated? Included in guidelines? What is the remuneration/payment structure for their use?

Why CRP testing brought in?

- Why was CRP testing introduced?
- Was this based on evidence?
- What evidence persuaded you?
- How quickly was the technology adopted?
- Was adoption universal/led by KOLs?
- How easily did others follow?
- Who pays for the test?

Perceived benefits

- What are the benefits of POC CRP testing?
- To patients?
- To clinicians? Incentives?
- To society/payers/healthcare system?
- What are the disadvantages?

Barriers and Facilitators

- How easy was it for the test to become adopted?
- What helped this? Why?
- What barriers were there to adoption of the test? Why?
- Explore: Financial/clinical/cultural/organisational/patient safety/regulation/design
- Quality assurance process

Which stakeholder groups were involved and their attitudes?

- Who are the main stakeholders in the use of POC CRP?
- Who is advantaged by the use of the test?
- Are any parties disadvantaged by the use of the test?

Overview of pathway and how it has changed with POC CRP

- How are patients with suspected LRTI managed?
- Where to these patients present?
- Explore clinical pathway, are changes specific to certain stakeholders?
- How has POC CRP changed the clinical pathway?
- Who performs the test? Who is responsible for acting on test result?
- Has it affected outcome?
- Has it affected hospital admissions?
- Has it affected antibiotic prescribing?
- Has it affected clinic flow? Patient engagement with health services?
